# Historic redlining and the siting of oil and gas wells in the United States

**DOI:** 10.1038/s41370-022-00434-9

**Published:** 2022-04-13

**Authors:** David J. X. Gonzalez, Anthony Nardone, Andrew V. Nguyen, Rachel Morello-Frosch, Joan A. Casey

**Affiliations:** 1 School of Public Health, University of California, Berkeley, Berkeley, CA USA; 2 Department of Environmental Science, Policy and Management, University of California, Berkeley, Berkeley, CA USA; 3 University of California (UC) Berkeley-UC San Francisco (UCSF) Joint Medical Program, UC Berkeley School of Public Health and UCSF School of Medicine, Berkeley and San Francisco, CA USA; 4 Department of Environmental Health Sciences, Columbia University Mailman School of Public Health, New York, NY USA

**Keywords:** Environmental justice, Geospatial analyses, Population based studies

## Abstract

**Background:**

The presence of active or inactive (i.e., postproduction) oil and gas wells in neighborhoods may contribute to ongoing pollution. Racially discriminatory neighborhood security maps developed by the Home-Owners Loan Corporation (HOLC) in the 1930s may contribute to environmental exposure disparities.

**Objective:**

To determine whether receiving worse HOLC grades was associated with exposure to more oil and gas wells.

**Methods:**

We assessed exposure to oil and gas wells among HOLC-graded neighborhoods in 33 cities from 13 states where urban oil and gas wells were drilled and operated. Among the 17 cities for which 1940 census data were available, we used propensity score restriction and matching to compare well exposure neighborhoods that were similar on observed 1940 sociodemographic characteristics but that received different grades.

**Results:**

Across all included cities, redlined D-graded neighborhoods had 12.2 ± 27.2 wells km^−2^, nearly twice the density in neighborhoods graded A (6.8 ± 8.9 wells km^−2^). In propensity score restricted and matched analyses, redlined neighborhoods had 2.0 (1.3, 2.7) more wells than comparable neighborhoods with a better grade.

**Significance:**

Our study adds to the evidence that structural racism in federal policy is associated with the disproportionate siting of oil and gas wells in marginalized neighborhoods.

## Introduction

Drilling and operating oil and gas wells in residential neighborhoods exposes residents to air and water pollution, noise, and other sources of stress that can increase risk of many types of disease [1–8]. An estimated 17 million U.S. residents live within 1.6 km (1 mile) of at least one active oil or gas well, meaning that there is widespread risk of exposure to air pollutants, hazardous chemicals, and other stressors [9]. Prior studies have found higher atmospheric concentrations of ozone, fine particulates, and volatile organic compounds downwind of oil and gas wells, and contamination of ground and surface waters near wells by dissolved solids and organic compounds [3, 4, 6, 8]. Researchers have also found that living near oil and gas wells is associated with higher risk of cardiovascular disease, impaired lung function, anxiety, depression, preterm birth, and impaired fetal growth [1, 5, 10–21]. In several studies, risk was heightened among racially and socioeconomically marginalized people, and in several U.S. regions these same groups have disproportionately high exposure to wells and natural gas flaring [12, 22–26]. Despite widespread exposures and evidence of racialized health disparities, the structural processes that shape these disparities are not well understood.

Current disparities in exposure to oil and gas wells could be driven by historic and persistent racist policies in housing, lending, and urban planning policies, including through a process known as redlining [27–29]. Over the past century, redlining has been perpetuated by both private businesses and government agencies, including the federal Home Owners Loan Corporation (HOLC) [28, 30]. The U.S. federal government first created HOLC to provide relief to mortgage holders through a federal lending program, with the aim of preventing mortgage foreclosures during the Great Depression [28]. Later in the 1930s, HOLC was also tasked with appraising and mapping neighborhood-level real estate risk in the 239 U.S. cities with populations over 40,000 [31]. In appraising mortgage risk, HOLC staff considered neighborhood level characteristics that included home values, whether there were industrial facilities, and the presence racially marginalized populations such as Black people and immigrants [28]. Neighborhoods that HOLC staff considered most “desirable” received a grade of A and were shaded green on HOLC maps; grade B neighborhoods were described as “still desirable” and shaded blue; C-graded neighborhoods (“definitely declining”) were shaded yellow; and D-graded neighborhoods (“hazardous”) were shaded red (i.e., redlined). Previous studies have established that, even controlling for housing value and the condition of housing stock, HOLC staff gave worse grades to neighborhoods based on the presence of Black and immigrant residents [31].

For researchers, HOLC maps remain relevant as indicators of the geographic extent of historic redlining policies, which have contributed to persistent racial residential segregation and health disparities in major U.S. cities [28, 31]. In fact, several studies have found that people living in neighborhoods that received poorer HOLC grades have higher risk of asthma, cancer, cardiovascular disease, gun violence-related injuries, heat-related emergency department visits, COVID-19 infection and mortality, preterm birth, and low birthweight [29, 32–41]. Additionally, people living in neighborhoods with worse HOLC grades have less access to environmental benefits such as greenspace and experience higher summertime temperature extremes [42, 43]. Residents of communities living near oil and gas wells have long called attention to racial and socioeconomic disparities in the siting of oil and gas wells in urban settings, even before the Great Depression [44]. However, it is unclear whether historical redlining processes were influenced by, or subsequently influenced, the siting of oil and gas wells in major U.S. cities. Accordingly, we sought to assess whether neighborhoods that received poorer HOLC grades have a disproportionately high number of wells, compared to higher graded neighborhoods.

## Methods

### Study design

Using digitized HOLC appraisal maps provided by the Mapping Inequality Project at the University of Richmond [45], and a national dataset of oil and gas wells dating back to 1898 [46], we conducted a retrospective assessment of the association between HOLC grade and the presence of oil and gas wells in 33 U.S. cities. Our overarching objective was to determine whether worse HOLC grades were associated with exposure to more oil and gas wells. To address this objective, we conducted a national cross-sectional analysis of the association between HOLC grades and siting of oil and gas wells drilled at any time since record-keeping began. We next conducted two secondary analyses that leveraged temporal variation in the siting of oil and gas wells. Specifically, we investigated whether the presence of oil and gas wells before HOLC appraisal occurred was associated with subsequently receiving a worse grade. We then examined whether receiving a worse HOLC grade was associated with subsequent disproportionate siting of wells.

**Fig. 1:**
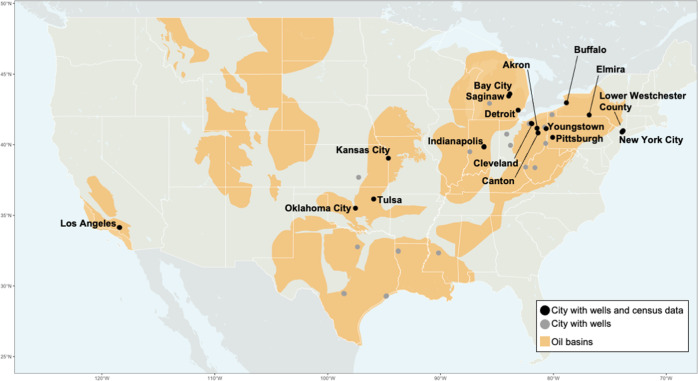
Map of cities included in the study, including cities with 10 or more wells within 100 m of neighborhoods appraised by the Home Owners Loan Corporation (HOLC). All cities included in the study were appraised by HOLC and census tract-level data from the 1940 census were available for a subset of 17 cities (labeled).

We included 33 cities in our analysis based on two criteria: first, that HOLC security maps were available; and second, that the city had at least 10 wells drilled or operated at any time within 100 m of HOLC-graded neighborhoods (Fig. 1). For the propensity score-matched analyses, we further restricted our analysis to those cities where tract-level data from the 1940 U.S. decennial census were available (*n* = 17).

### Data

We obtained digitized HOLC maps from the Mapping Inequality project at the University of Richmond [45]. The full dataset includes data for 198 cities in 38 states, digitized from original paper maps produced by HOLC and including original neighborhood boundaries and grades. We obtained census-tract level geospatial and sociodemographic data from the 1940 census from the National Historical Geographic Information System from Integrated Public Use Microdata Series [47]. The 1940 census included census tract-level data for 60 cities, for which HOLC risk maps were also available (Supplementary Table S1). For each HOLC neighborhood where 1940 census tract-level data were available (*n* = 1695), we used areal apportionment to assign tract-level sociodemographic characteristics to HOLC neighborhoods, as described previously by Nardone et al. [42] (Supplementary Fig. S1).

Data on oil and gas wells dating back to 1898 were obtained from Enverus DrillingInfo, a private service that aggregates oil and gas operational data from operators and state agencies across the U.S., primarily for use in industry and made available for research purposes [46]. We assembled data for all wells within 1 km of any HOLC-graded neighborhood. The analytic dataset includes geographic coordinates, spud date (when the preproduction phase begins), completion date (when preproduction ends), first production date, last production date, and well type (oil, gas, oil and gas, injection, or unknown). We included oil, gas, oil and gas, injection, and unknown well types in our analyses.

### Exposure assessment

**Fig. 2:**
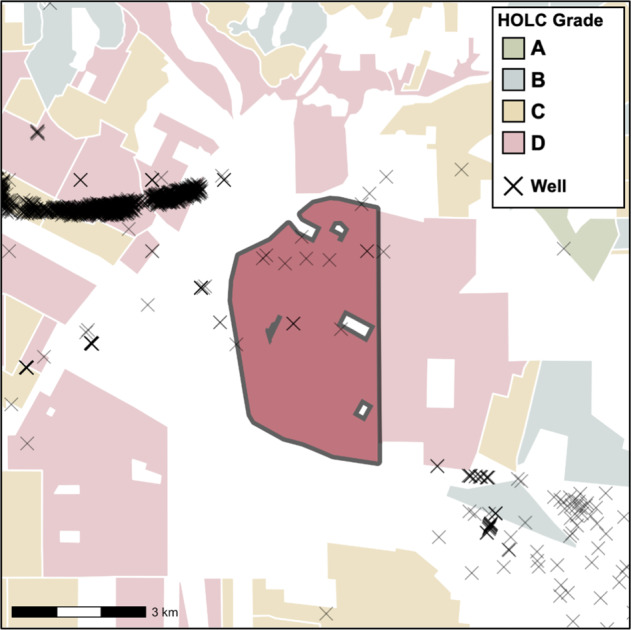
Illustration of the exposure assessment protocol with data from neighborhood D53 in Los Angeles, California. For each HOLC-graded neighborhood, we counted wells within 100 m of the neighborhood boundary (gray outline).

For all analyses, the geographic unit of observation was the HOLC-graded neighborhood. The neighborhood boundaries were defined by HOLC appraisers to comprise areas that were demographically homogenous and had similar housing characteristics, rather than to correlate with administrative or political boundaries [31]. For each neighborhood, we first assessed cumulative exposure to all oil, gas, oil and gas, injection, or unknown wells sited at any time within 100 m of the neighborhood boundary (Fig. 2). We did this for wells drilled or operated at any time, including wells without available production dates. Our aim was to count wells both inside and just outside of neighborhoods that could have adverse effects for residents. We chose to use a 100 m buffer rather than larger buffers so as not to subsume adjacent HOLC-graded neighborhoods, some of which had relatively small areas (the minimum area among neighborhoods with wells was 0.08 km^2^). The exposure assessment included all well types in aggregate and separately for each well type (i.e., oil, gas, oil and gas, injection, and unknown) (Supplementary Fig. S2).

In secondary analyses, we also assessed exposure to wells drilled before and after HOLC appraisal occurred in each city (Supplementary Fig. S3). In the pre-appraisal assessments, we counted wells that had at least one production date before the date of appraisal in each city (e.g., spud date or first date of production was in 1934 or earlier for a city appraised in 1935). Similarly, for the post-appraisal analyses, we counted wells drilled or operated after appraisal occurred in each city through 2021, excluding wells with any production date before appraisal occurred. Wells where no dates were provided (i.e., missing spud, completion, and first or last production dates) were, for obvious reasons, excluded from the pre- and post-HOLC appraisal analyses as we could not determine whether these wells were drilled before or after appraisal. We did, however, include wells without any reported dates in the primary analyses along with wells with reported dates.

### Propensity score restriction and matching

We used propensity score matching to control for confounding by identifying neighborhoods that received different grades, but that were comparable in terms of observed sociodemographic factors [48]. For example, among neighborhoods that received a C grade, some may have comparable sociodemographic characteristics to neighborhoods that received a D grade, whereas others may have had substantially different sociodemographic characteristics. We may infer, then, that C-graded neighborhoods that have similar sociodemographic characteristics to D-graded neighborhoods are more exchangeable than C-graded neighborhoods that were substantially different and had little probability of being redlined. By exchangeable, we mean that, e.g., the matched C-graded neighborhoods more closely approximate what conditions in the D-graded neighborhoods would have been like had the D-graded neighborhoods instead received a grade of C.

For the subset of neighborhoods with apportioned 1940s census data (*n* = 1695 in 17 cities), we estimated propensity scores using observed 1940s sociodemographic characteristics apportioned from census tracts to HOLC-defined neighborhoods. We aimed to identify neighborhoods with adjacent grades (i.e., D and C, C and B, and B and A) and similar observed characteristics. This approach is similar to the methods described in Nardone et al.’s investigations of the associations between redlining and adverse birth outcomes [33] and greenspace [34]. We included the following variables: total population; proportion of Black, foreign-born, and non-White employed residents; proportion of residents who had completed high school; number of homes; median value homes; proportion of homes in need of major repair; proportion of homes with radios; and number of people in the home. We included the non-White category because the 1940 census did not further disaggregate racial groups.

To estimate the propensity scores, we used an ensemble of machine learning algorithms (generalized linear models [GLM], Bayesian GLM, multivariate adaptive regression splines, and generalized additive models [GAMs]), using the SuperLearner package in R, as described previously by Nardone et al. [42, 49]. In estimating propensity scores, we used all variables mentioned above as covariates. We conducted pairwise comparisons between neighborhoods that received adjacent grades, i.e., comparing neighborhoods graded A to those graded B, B to C, and C to D (Supplementary Fig. S4). The resulting scores describe the likelihood of receiving the treatment; in this case, the lower respective HOLC grade. Based on propensity scores, we omitted neighborhoods that did not have a propensity to receive the adjacent grade. Specifically, we excluded neighborhoods that had scores above the 99^th^ percentile to receive the higher respective grade or below the 1^st^ percentile to receive the poorer grade (*n* = 788). We did this to exclude neighborhoods that, based on observed sociodemographic characteristics, were highly unlikely to receive a different grade. With the remaining neighborhoods, we matched neighborhoods with adjacent HOLC grades to the nearest propensity score-neighbor, with replacement.

### Statistical analyses

We first described the spatial distribution of wells in HOLC-graded neighborhoods, including wells sited at any time and those sited before and after HOLC appraisal occurred. We calculated summary statistics comparing the number of wells within 100 m of neighborhood boundaries, and well count normalized by area (i.e., wells per km^2^). We used analysis of variance (ANOVA) to determine whether there were statistically significant differences in the number or density of wells between neighborhoods with varying HOLC grades. We included all 8,908 neighborhoods graded by HOLC in cities with 10 or more wells for this set of cross-sectional cumulative well analyses.

Next, we aimed to determine whether worse HOLC grades were associated with the presence of wells drilled or operated at any time in neighborhoods. For these analyses, we used the propensity score-matched datasets. Since we relied on census data to construct the propensity scores, analyses were restricted to the 17 cities with available 1940 census tract-level data. After implementing propensity score restriction and mapping as described above, we used targeted maximum likelihood estimation (TMLE) to determine whether higher exposure to wells (of any type) was associated with worse HOLC grades. We did this for each pairwise comparison: A- versus B-graded neighborhoods, B- versus C-graded neighborhoods, and finally C- versus D-graded neighborhoods.

As a sensitivity analysis, we fit models with exposure to wells fully contained within each HOLC neighborhood (i.e., without the 100 m buffer). Additionally, because neighborhoods ranged widely in size, we also conducted sensitivity analyses where the exposure metric was well density within 100 m of the HOLC boundaries (number of wells per km^2^) instead of well count.

Finally, in a set of secondary analyses, we took advantage of temporal variation in the siting of wells. For these analyses we were restricted to wells with drilling or production dates. We used TMLE to assess the association between the number of wells and HOLC grade, but with exposure limited to those wells that were drilled or operated before appraisal occurred. Our aim was to determine whether the presence of wells prior to HOLC appraisal was associated with subsequently receiving a worse grade. We then used TMLE to examine the association between HOLC grade and count of wells drilled or operated after appraisal. Our aim here was to determine whether receiving a worse HOLC grade was associated with subsequent exposure to more wells.

We used an alpha of 0.05 to determine statistical significance. All data preparation and analyses were conducted using R v 4.0 [50].

### Ethical considerations

All sociodemographic data used in this analysis were publicly available and aggregated; we did not use any data from human subjects.

## Results

### Sociodemographic data

The descriptive analyses included data from 33 cities in 13 states (Fig. 1). Cities included in the analytic dataset were appraised between 1935 and 1940. In these cities, there were a total of 2497 HOLC-graded neighborhoods, of which 735 were exposed to at least one well within 100 m. The average area of all neighborhoods across these cities was 1.41 ± 2.40 km^2^ (range: 0.02, 66.8). Among exposed neighborhoods, the mean area was 2.20 ± 3.76 km^2^ (range: 0.07, 37.5).

Tract-level data from the 1940 census were available for 60 cities from 25 states, with a total of 3408 HOLC-graded neighborhoods across these cities. Of these cities, 17 had at least 10 wells within 100 m of a HOLC-graded neighborhood and were included in analyses (Supplementary Table S1). These cities were: Los Angeles, CA; Cleveland, OH; Oklahoma, OK; Canton, OH; Akron, OH; Saginaw, MI; Tulsa, OK; Youngstown, OH; Kansas City, MO; New York City, NY; Detroit, MI; Indianapolis, IN; Pittsburgh, PA; Bay City, MI; Buffalo, NY; Lower Westchester County, NY; and Elmira, NY.

Data from the 1940 census were available for a total of 1,695 HOLC-graded neighborhoods in the 17 cities with wells; these comprised our dataset for the propensity score-matched analyses. The total estimated 1940 population of these neighborhoods was 14,549,910, approximately 11% of the total 1940 U.S. population (Table 1). Worse HOLC grades were associated with lower median home value and lower educational attainment. D-graded neighborhoods that received D grades from HOLC had disproportionately high populations of Black, non-White, or foreign-born residents. Fewer people in D-graded neighborhoods had completed high school than residents of neighborhoods graded A or B. Across all cities, approximately 65% of Black people lived in D-graded neighborhoods. Residents of D-graded neighborhoods were also less likely to live in owner-occupied housing or to have radios in the household than residents of neighborhoods that received other grades.

**Table 1:** Population characteristics from the 1940 U.S. Census stratified by HOLC grade. These data are for the 17 cities that were appraised by HOLC, had at least ten oil or gas wells within 100 m of a graded neighborhood, and had digitized 1940s census tracts data. Note that the demographic variables are not mutually exclusive. Median home value reported in US dollars with mean and standard deviation of neighborhood-level median value for each HOLC grade.

	HOLC Grade				
Variable	A	B	C	D	All
Neighborhoods, *n*	174	391	712	418	1695
Total area, km^2^	395.3	768.9	1951.2	1104.4	4219.8
Population, *n*	1,106,840	3,115,749	6,666,349	3,660,972	14,549,910
Population density, *n* km^−2^	2800.0	4052.2	3416.5	3314.9	3448.0
Demographics, *n* (%)					
Black	16,578 (1.5)	42,611 (1.4)	103,369 (1.6)	317,311 (8.7)	489,869 (3.3)
White	1,085,821 (98.1)	3,064,941 (98.4)	6,546,550 (98.2)	3,330,584 (91.0)	14,027,896 (96.4)
Non-White	21,016 (1.9)	50,785 (1.6)	119,792 (1.8)	330,354 (9.0)	521,947 (3.6)
Foreign-born	175,501 (15.9)	537,264 (17.2)	1,261,263 (18.9)	736,657 (20.1)	2,710,685 (18.6)
Completed high school, *n* (%)	192,547 (17.4)	455,292 (14.6)	720,113 (10.8)	287,362 (7.8)	1,655,314 (11.4)
Median home value, mean ± SD	7,603 ± 3,841	5,701 ± 2,670	4,382 ± 1,911	3,247 ± 2,140	4,737 ± 2,732
People per home, mean ± SD	3.3 ± 1.1	3.4 ± 0.9	3.1 ± 1.3	3.0 ± 1.1	3.2 ± 1.1
Owner-occupied housing, %	44.2	39.9	40.3	30.3	38.1
Radio ownership, %	95.4	95.4	95.0	92.2	94.4

### Exposure to wells

We obtained data for 12,060 wells sited between 1898 and 2021 that were within 100 m of at least one HOLC-graded neighborhood. Most of the wells (78.2%) were oil or gas wells, 2.1% were injection wells, and 19.7% were of unknown type. Among all wells, 51.0% had production dates (spud date, completion date, or first or last date of production). Some 10,124 wells were inside neighborhood boundaries defined by HOLC and an additional 1936 were located within 100 m of neighborhood boundaries. Los Angeles, California, had the most wells with 6618 of all types within 100 m of the boundaries of HOLC-graded neighborhoods, followed by Cleveland, Ohio, with 1193 and Oklahoma City, Oklahoma, with 1167 (Supplementary Table S1).

**Fig. 3:**
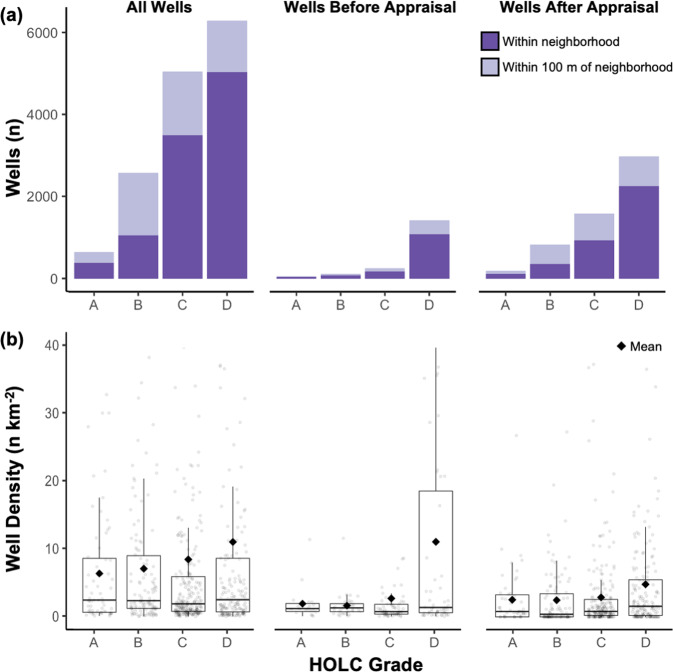
Distribution of exposure to wells by HOLC neighborhood grade. **a** Count of wells inside HOLC neighborhoods or within 100 m of the neighborhood boundary, stratified by HOLC grade. We considered exposure to all wells (left, including those without production dates), and wells drilled or operated before (middle) and after (right) appraisal occurred in each city. Note that approximately half of wells did not have production dates, and we were unable to include this in the before or after appraisal figures. **b** Density of wells per square km among HOLC neighborhoods with any exposure, stratified by grade. Each point represents the well density in one neighborhood. Similarly, we considered all wells (left) and wells drilled or operated before (middle) or after (right) appraisal occurred. The *y*-axis for panel **b** is restricted to 0–40 wells km^−2^ to better illustrate summary statistics, though several neighborhoods had densities above this range.

Across all cities, worse HOLC grades were associated with exposure to successively more wells located inside or nearby the neighborhood boundaries (Fig. 3a). A-graded neighborhoods had 647 wells within 100 m, B-graded neighborhoods had 2581 wells, C neighborhoods had 5051, and D-graded neighborhoods had 6288 wells. For wells drilled or operated before HOLC appraisal, neighborhoods that went on to receive a D grade had more wells (*n* = 1421) than all other graded neighborhoods combined (*n* = 440). After appraisal occurred, A-graded neighborhoods had 192 wells, B neighborhoods had 835 wells, C neighborhoods had 1590 wells, and redlined D-graded neighborhoods had 2,977 wells sited. Within the most exposed cities, we generally saw more wells in neighborhoods with worse grades (Supplementary Fig. S5). In Los Angeles and Oklahoma City, most wells were in D-graded cities. In San Antonio and Cleveland, C-graded neighborhoods had the most wells. When we disaggregated wells by type, the majority of oil gas wells and injection wells were in D-graded neighborhoods (Supplementary Fig. S6). Most wells of unknown type were in C-graded neighborhoods.

There were 6089 wells with no reported production dates located within 100 m of HOLC neighborhood boundaries. Some 5403 wells were within neighborhoods boundaries, and most of these were in neighborhoods graded C (44.0%) or D (31.4%). Only 4.1% were in A-graded neighborhoods. Nearly half (49.4%) of wells sited inside HOLC-defined neighborhoods that had missing dates were in Los Angeles, where the majority were in D-graded neighborhoods.

Among neighborhoods with any exposure, the mean density of wells per km^2^ was higher in those with worse HOLC grades (Fig. 3b). Redlined neighborhoods had an average of 12.2 ± 27.2 wells km^−2^, nearly twice the density of A-graded neighborhoods at 6.8 ± 8.9 wells km^−2^, though the difference was not statistically significant. There was a significantly higher density of wells per km^2^ sited before HOLC appraisal in D-graded neighborhoods than neighborhoods with other grades (*p* < 0.001). There was a mean of 14.1 ± 24.9 wells km^−2^ in D-graded neighborhoods pre-HOLC, with averages of 2.5–2.8 wells km^−2^ in A- through C-graded neighborhoods. Post-appraisal, there were significantly higher density of wells in redlined neighborhoods (6.5 ± 11.1 wells km^−2^ in D-graded compared to 3.7–4.8 wells km^−2^ in A-C graded neighborhoods). Among the subset of cities with 1940 census data, we observed a higher density of wells sited before appraisal in D-graded neighborhoods (3.7 ± 10.5 wells km^2^) compared to A-graded neighborhoods (2.4 ± 4.3), though the difference was not statistically significant. Median well densities were similar across HOLC grades, indicating that the significantly higher mean densities in neighborhoods with worse grades were attributable to a subset with high exposure.

### Propensity score restricted and matched analyses

**Fig. 4:**
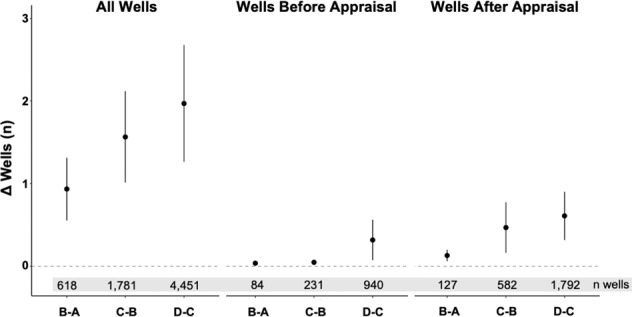
Point estimates and 95% confidence intervals for the difference in the number of wells within 100 m of neighborhood boundary, comparing neighborhoods with adjacent HOLC grades. These points represent the estimated increase in well count for neighborhoods with the relatively worse HOLC grade compared to propensity score-matched neighborhoods with the relatively better grade (e.g., estimated additional wells in D-graded neighborhood compared to matched C-graded neighborhoods). We conducted separate analyses for: all wells, including those without production dates (left); wells drilled or operated before HOLC appraisal occurred in each city (middle); and wells drilled or operated after HOLC appraisal occurred (right). The number of wells neighborhoods were exposed to in each model are reported above the *x*-axis.

Across all comparisons, neighborhoods with worse HOLC grades had significantly more wells (Fig. 4). Neighborhoods graded B had 0.9 (95% CI: 0.6, 1.3) more wells than propensity score-matched neighborhoods graded A. We also observed that C-graded neighborhoods had 1.6 (1.0, 2.1) more wells than B-graded neighborhoods. Redlined D-graded neighborhoods had 2.0 (1.3, 2.7) more wells than C-graded neighborhoods. Before HOLC appraisal occurred, D-graded neighborhoods had 0.3 (0.1, 0.6) more wells than C-graded neighborhoods. After appraisal occurred, we also found that neighborhoods with worse grades had significantly more wells. Our findings did not change when we used the exposure metric restricted to wells inside each neighborhood (Supplementary Fig. S7). The results were also similar when we used well density instead of well count as the exposure metric (Supplementary Fig. S8).

## Discussion

We investigated whether historic redlining was associated with higher exposure to oil and gas wells. We found more wells and a higher density of wells in neighborhoods that received worse HOLC grades. In propensity score restricted and matched analyses, we observed significantly more wells in neighborhoods with worse HOLC grades. These findings were robust to sensitivity analyses.

Residents of historically redlined neighborhoods located in cities with oil and gas production have disproportionately high exposure to oil and gas wells, compared to higher graded neighborhoods. We observed more wells and higher mean density of wells per km^2^ in neighborhoods with successively worse grades, overall and before and after appraisal occurred in each city. Redlined neighborhoods, or those graded D by HOLC, consistently had the most wells. In propensity score restricted and matched analyses, we found that neighborhoods with worse HOLC grades had significantly more wells. The largest difference was in D-graded neighborhoods, which had approximately two more wells than matched C-graded neighborhoods. Our findings add to the evidence that the legacy of structural racism through redlining is associated with higher oil and gas well exposures. The disparities were consistent across well type, which is notable given that the environmental hazards and health risks associated with oil and gas wells may be different than those associated with oil and gas wells; risks may also vary between conventional and unconventional wells. These exposure disparities have implications for community environmental health, as the presence of active and abandoned (i.e., postproduction) wells have been shown to contribute to ongoing air pollution [4, 51, 52].

Disproportionate exposure to oil and gas wells could contribute to health disparities. Recent population health studies have reported that racially and socioeconomically marginalized people had disproportionately high risk of adverse birth outcomes with exposure to oil and gas production facilities. Gonzalez et al. [12] observed that exposure to oil and gas production was associated with higher risk of spontaneous preterm birth among parents in the San Joaquin Valley, California, and that the risk was confined to parents who were Hispanic, Black, or had not completed high school. A 2022 meta-analysis by Lee et al. found significantly higher risk of preterm birth in historically redlined neighborhoods compared to non-redlined neighborhoods, and oil and gas wells have been found to emit air pollutants associated with preterm birth risk [4, 29]. These findings, in aggregate with the present study, indicate one plausible biological pathway through which redlining could increase preterm birth risk. In a study of exposure to natural gas flaring from oil and gas wells in Texas, Cushing et al. [24] found that the risk of adverse birth outcomes was higher among Hispanic parents compared to non-Hispanic White parents. In Pennsylvania, Casey et al. reported increased risk of anxiety and depression during pregnancy among those living close to more wells, a relationship that was stronger among individuals relying on Medicaid [53]. People living in neighborhoods with higher well density are exposed to higher concentrations of chemical contaminants and other stressors associated with oil and gas production, such as heavy traffic and noise [2, 7, 54, 55]. Racially and socioeconomically marginalized people, therefore, may have suffered disproportionately high exposure to oil and gas-related contaminants attributable to 80-year-old racist policies related to mortgage risk assessments and housing discrimination. This phenomenon warrants further study, to elucidate the role of these structural determinants on community environmental health and health disparities.

Our study advances scientific understanding regarding the role of redlining in shaping the disproportionate exposure of marginalized communities to upstream oil and gas infrastructure in urban areas. Archival work by Cumming [56] showed that HOLC staff in Los Angeles considered the presence of oil and gas wells when conducting their appraisals and drawing their risk maps. Cumming also found that the racial composition of neighborhoods affected whether appraisers downgraded neighborhoods that contained wells. Specifically, the presence of wells was not treated as a penalizing factor in predominantly white neighborhoods with racially restrictive covenants, but it was a penalizing factor in neighborhoods with relatively high non-white populations [56]. This process could account, in part, for the disparities we observed in Los Angeles and elsewhere. However, it is unclear whether the presence of wells was considered by HOLC appraisers in other U.S. cities. There are several potential explanations for our finding that wells were already present in racially marginalized neighborhoods before HOLC appraisal occurred, including racist policies that pre-dated and correlated with HOLC-defined redlining. Redlining by HOLC and other agencies and firms may have contributed to the perpetuation of existing racialized disparities in exposure to wells.

The HOLC maps reflect one of many methods through which area-level racial housing discrimination was practiced in the 1930s and 1940s [28]. Other studies have found that racially and socioeconomically marginalized populations have disproportionately high exposure to oil and gas wells, including in places with and without HOLC maps. In a study of wastewater injection wells in Ohio, Silva et al [22] found that median income was inversely associated with the presence of wells. Johnston et al. [23] found that Hispanic residents in the Eagle Ford Shale, a rural region in central Texas, have disproportionately high exposure to natural gas flaring compared to their white counterparts. Similarly, assessments from the Natural Resources Defense Council and FracTracker Alliance report that that racially and socioeconomically marginalized communities have disproportionately high exposure to oil and gas wells in Los Angeles and Kern Counties, in California [25, 26]. These findings other factors of structural racism warrant further study with respect to the siting of oil and gas infrastructure.

This study was constrained by available data. The 1940 decennial census did not provide disaggregated data for many groups that faced racialized housing segregation in the early twentieth century, such as Mexican-American and Chinese-American residents of Los Angeles [57]. Census tract data were not available for many cities with both HOLC grades and wells (*n* = 16), including several of the cities with the most exposure to wells such as San Antonio, Texas, and Erie, Pennsylvania. In San Antonio, for example, many more wells were sited in C-graded neighborhoods than all other neighborhoods. However, because 1940 census data were not available, we excluded southern U.S. cities in Texas, Louisiana, and Mississippi from the propensity score-matched analyses. In 1940, Black people comprised 14.4% of the population of Texas, 35.9% of the population of Louisiana, and 49.2% of the Mississippi population [58]. The exclusion of cities in the southern U.S., a region where racialized segregation has historically been extreme, may have led to underestimate of the association between redlining and wells. Additionally, there was substantial missingness with respect to oil and gas well production dates. This may have led to misestimation of the association between HOLC grades and the siting of wells both before and after appraisal, though this would not be an issue for the analyses including all wells. The missingness of production dates may be associated with older wells, possibly due to difficulties of record keeping in the early twentieth century. If that were the case, it could have resulted in an undercount of the number of wells in C- and D- graded neighborhoods (where most of these wells were located) prior to appraisal. We were not able to investigate cities with oil and gas wells that were not appraised by HOLC, but where other types of area-level housing and mortgage discrimination occurred. There may also be residual confounding not accounted for in the propensity score restriction and matching procedure. The HOLC maps were just one of several forms of racist housing segregation employed by government agencies and private firms. These maps capture redlining at a cross-section from the perspective of HOLC appraisers but are not exhaustive and may not reflect redlining practices in other times and in other places, or even concurrent redlining practices by other agencies and firms. We also did not consider modern forms of housing segregation, such as predatory lending and racialized disparities in housing appraisal.

Our study benefitted from a broad scope with national-level data, including digitized HOLC maps from the University of Richmond. The propensity score matching approach allowed us to better control for confounding and to more accurately estimate the relationship between siting of wells and subsequent redlining and between redlining and the subsequent siting of wells. Our findings were consistent across all statistical analyses and models, and the results were robust to sensitivity analyses.

We conducted a retrospective assessment of the association between grades assigned on racially discriminatory neighborhood security maps from the federal Home Owners Loan Corporation and the siting of oil and gas wells. In neighborhoods that were comparable at the time of appraisal but that received a different HOLC grades, the neighborhoods with worse grades had a higher number and density of wells. The presence of wells in historically redlined neighborhoods remains relevant, as many of these redlined neighborhoods have persistent social inequities and the presence of wells, both active and post-production, can contribute to ongoing pollution. Our study adds to the evidence that structural environmental racism contributed to the disproportionate siting of oil and gas wells in racially and socially marginalized neighborhoods. In follow-up work, researchers may consider examining whether other environmental hazards are associated with historic redlining, as well as examining the potential effects of redlining policies not associated with HOLC.

## Supplementary information

Supplementary information

## Data Availability

The code and datasets supporting the conclusions of this article are available on GitHub at https://github.com/djxgonzalez/us-drilling-redlining.
