# Supplementary material for: Historic redlining and the siting of oil and gas wells in the United States
Source: J Expo Sci Environ Epidemiol. 2022 Apr 13;33(1):76–83. doi: 10.1038/s41370-022-00434-9 (PMC9556657; doi:10.1038/s41370-022-00434-9)
Supplement: Supplementary file 1 — Supplementary information [file 41370_2022_434_MOESM1_ESM.pdf]

Historic redlining and siting of oil and gas wells in the United States

David J.X. Gonzalez, Anthony Nardone, Andrew V. Nguyen, Rachel Morello-Frosch, and Joan A. Casey

Table S1. List of cities (n = 33) included in the analytic dataset with the count of all wells sited at any time inside HOLC neighborhoods (i.e., within 0 m buffer) and within 100 m of the boundaries of all HOLC-graded neighborhoods. In primary analyses, we included cities that had 10 or more wells within 100 m of HOLC-graded neighborhoods. For analyses with propensity score matching, we were constrained to cities where data from the 1940 decennial census was available (n = 17).

		1940 census	Wells in neighborhood (n)	
City	State	data available	Within	100 m buffer
Los Angeles	CA	Yes	5311	6618
Cleveland	OH	Yes	972	1193
San Antonio	TX	–	849	886
Oklahoma City	OK	Yes	846	1167
Erie	PA	–	396	996
Canton	OH	Yes	209	295
Akron	OH	Yes	187	245
Fort Worth	TX	–	185	264
Saginaw	MI	Yes	174	225
New York City	NY	Yes	134	154
Lima	OH	–	119	176
Tulsa	OK	Yes	86	155
Youngstown	OH	Yes	78	97
Grand Rapids	MI	–	72	99
Wichita	KS	–	70	120
Greater Kansas City	MO	Yes	64	158
Huntington	WV	–	53	71
Detroit	MI	Yes	45	142
Pittsburgh	PA	Yes	44	66
Lorain	OH	–	34	38
Shreveport	LA	–	32	76
Jackson	MS	–	25	49
Wheeling	WV	–	18	23
Springfield	OH	–	18	21
Galveston	TX	–	16	19
Charleston	WV	–	13	17

Lower Westchester Co.	NY	Yes	9	20
Warren	OH	–	8	29
Buffalo	NY	Yes	8	29
Elmira	NY	Yes	1	13
Indianapolis	IN	Yes	0	84
Bay City	MI	Yes	0	34
Terre Haute	IN	–	0	12

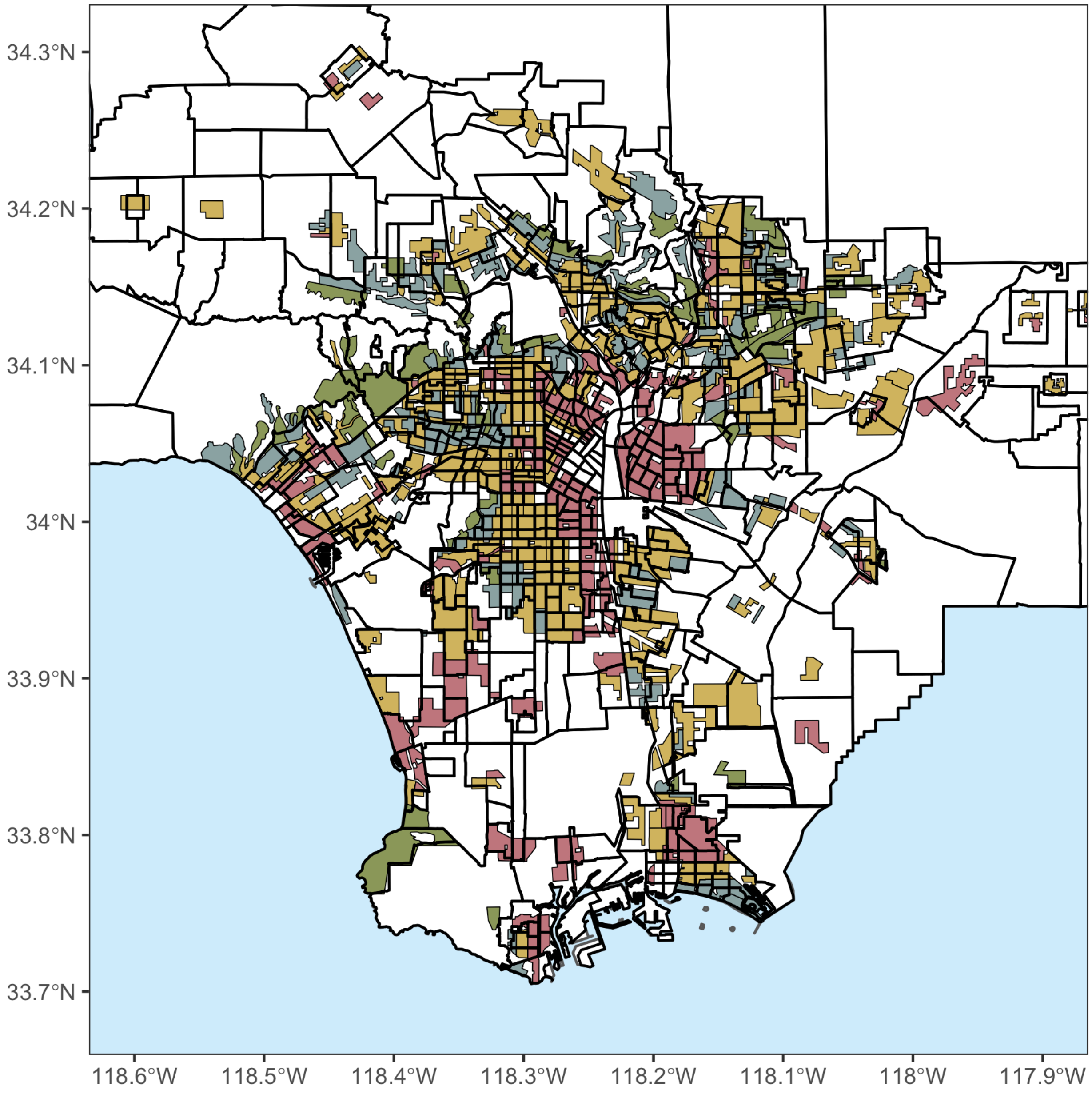

Figure S1. Map of 1940 census tracts boundaries (thicker lines) on top of graded HOLC neighborhoods (Grade A is green, B is blue, C is yellow, and D is red) in southern Los Angeles, California.

Cities	Neighborhoods	Wells	Research aims and analyses
**198 cities** with digitized HOLC maps from University of Richmond	**8,878 neighborhoods** graded by HOLC in these 198 cities	Obtained data for **22,600 wells** within 1 km of boundaries of HOLC-graded neighborhoods in these 198 cities	
Excluded cities with < 10 wells within 100 m of a HOLC-graded neighborhood			
**33 cities** with at least 10 wells within 100m	**2,497 neighborhoods** graded by HOLC in these 33 cities	**12,060 wells** within 100 m of these HOLC-graded neighborhoods drilled or operated at anytime from 1898 to 2021	Describe distribution of wells by HOLC grade in all cities graded by HOLC that had at least ten wells. We did this for all wells drilled at anytime and wells drilled before and after HOLC appraisal (Figs. 3, S5, S6).
Excluded cities without 1940 census tract data			
**17 cities** with census data	**1,695 neighborhoods** in these 17 cities	**8,741 wells** within 100 m of these neighborhoods	Determine whether neighborhoods that are comparable (based on observed 1940 census characteristics) but have worse HOLC grades have more wells (Figs. 4, S7, S8).
		Excluded wells without production dates	
**17 cities**	**1,695 neighborhoods**	**4,839 wells** drilled or operated before HOLC appraisal<br>**1,925 wells** drilled or operated after HOLC appraisal	First, to determine whether the presence of wells before HOLC appraisal occurred was associated with subsequently receiving a worse grade. Second, to determine whether receiving a worse HOLC grade weas associated with subsequent exposure to more wells (Figs. 4, S7, S8).

Figure S2. Flow chart showing exclusions (*italicized*) and the data used for each set of analyses.

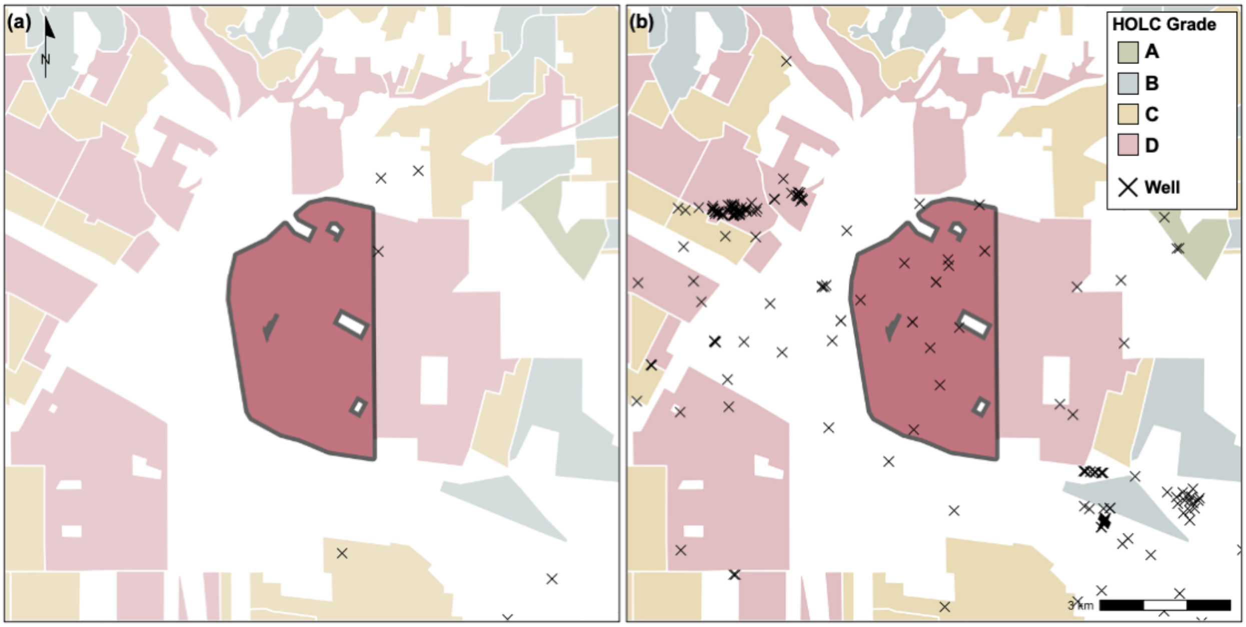

Figure S3. Illustrations of the exposure assessment protocol with real data from neighborhoods in Los Angeles, California. Neighborhood D53 is highlighted with the 100 m buffer drawn around it (gray shading). For each HOLC-graded neighborhood, we counted wells drilled or operated (a) before and (b) after HOLC appraisal occurred in each city.

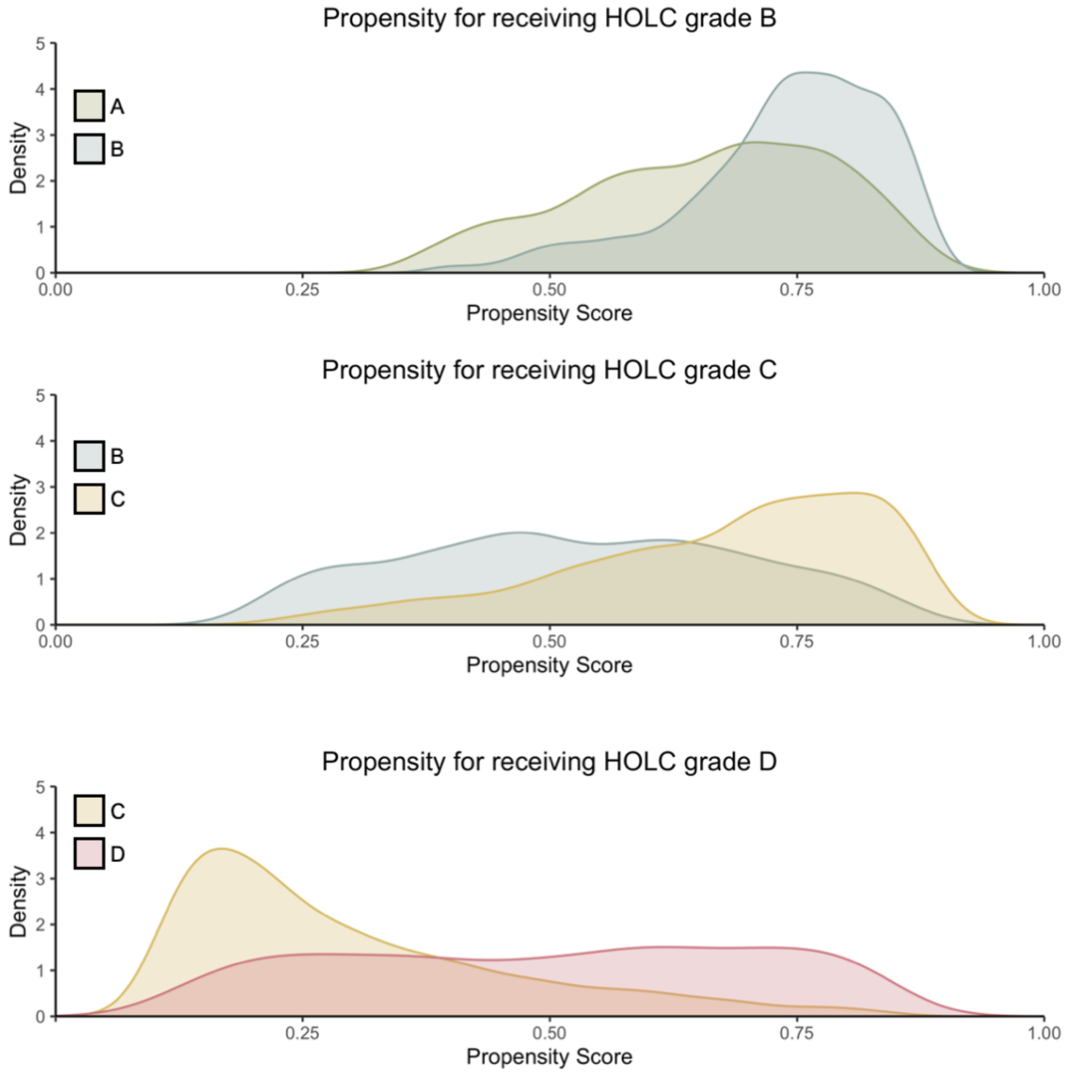

Figure S4. Propensity score distributions for neighborhoods graded by the federal Home Owners Loan Corporation (HOLC) with apportioned sociodemographic data from the 1940 U.S. decennial census.

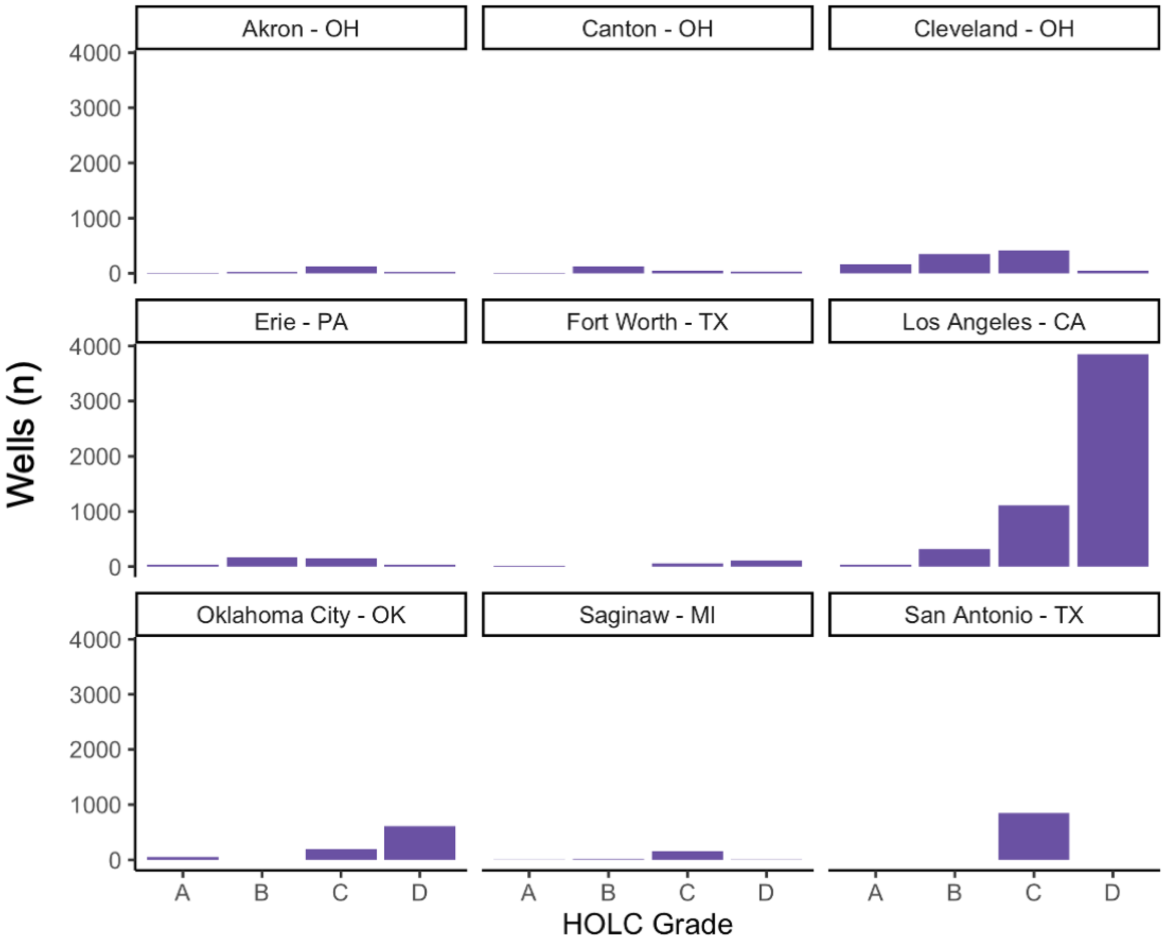

Figure S5. Count of all wells within 100 m of HOLC-graded neighborhoods, stratified by city. Restricted to the nine cities with the highest number of wells.

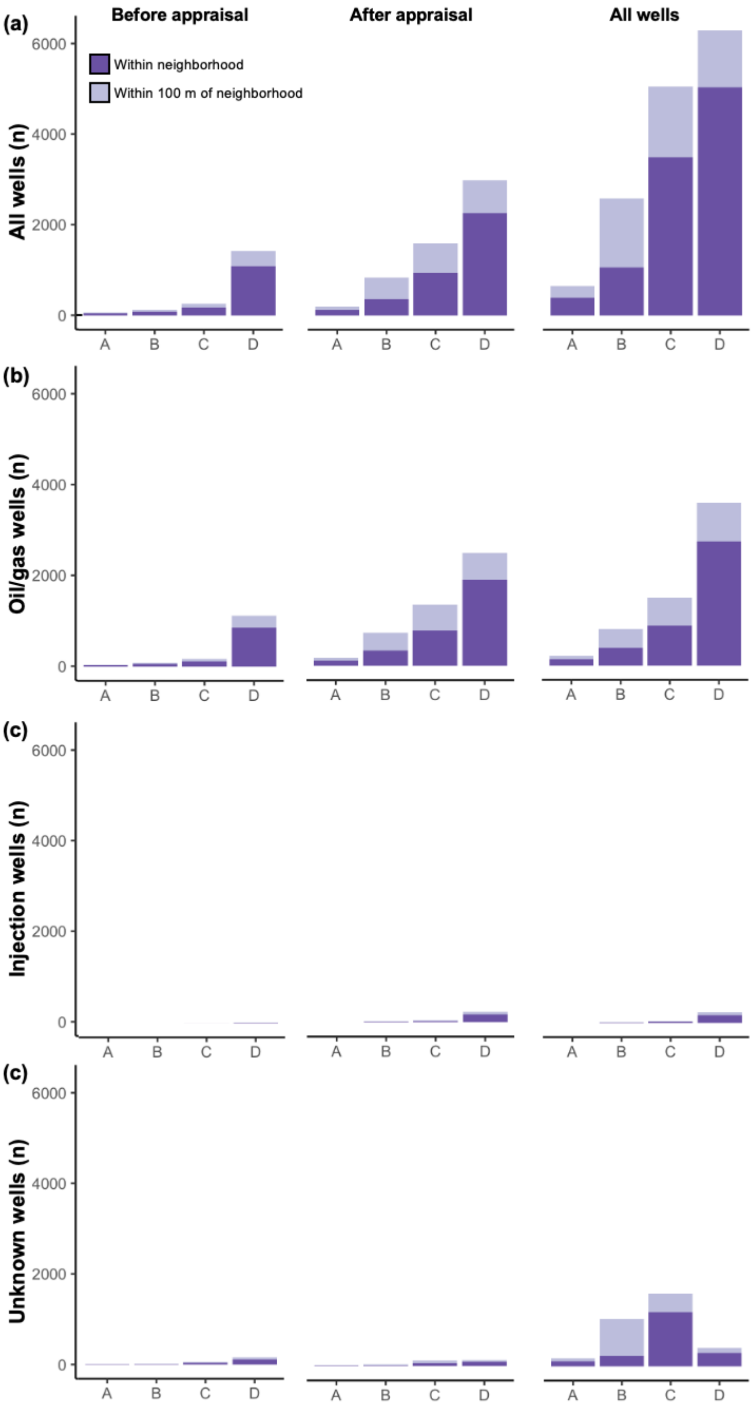

Figure S6. Distribution of wells by type stratified by HOLC grade. Panel (a) is the same as Figure 3a, included here for comparison.

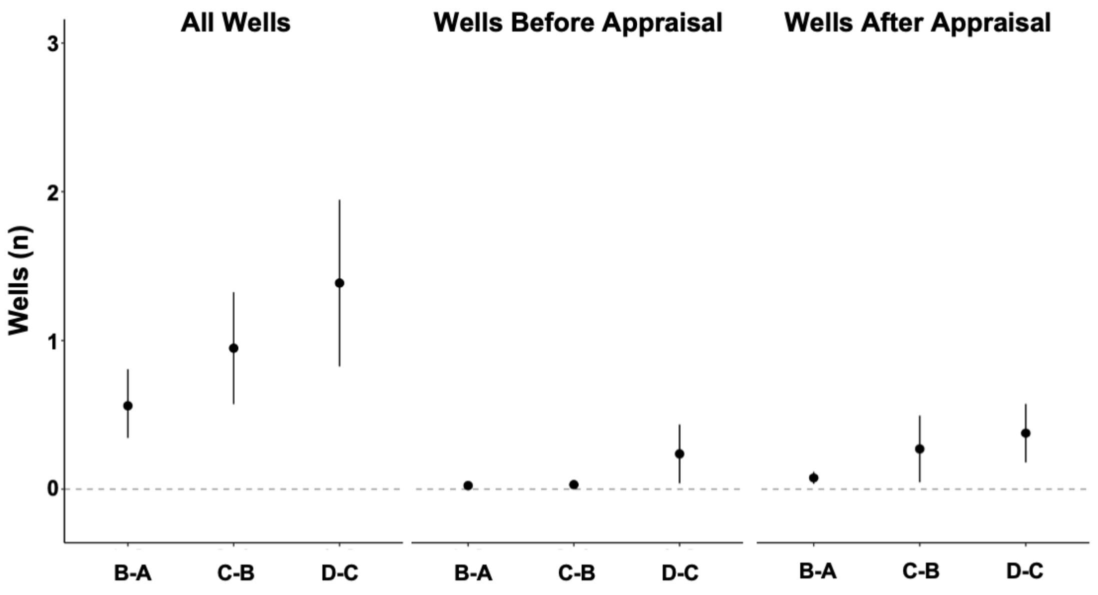

Figure S7. Point estimates and 95% confidence intervals for the difference in the number of wells inside neighborhood boundaries, comparing neighborhoods with adjacent HOLC grades. These points represent the estimated difference in well count for neighborhoods with the relatively worse HOLC grade compared to propensity score-matched neighborhoods with the relatively better grade. We conducted separate analyses for: (a) all wells, including those without production dates; (b) wells drilled or operated before HOLC appraisal occurred in each city; and (c) wells drilled or operated after HOLC appraisal occurred. These results are similar to those reported in Figure 4, except for wells inside neighborhood boundaries rather than wells within 100 m of neighborhood boundaries.

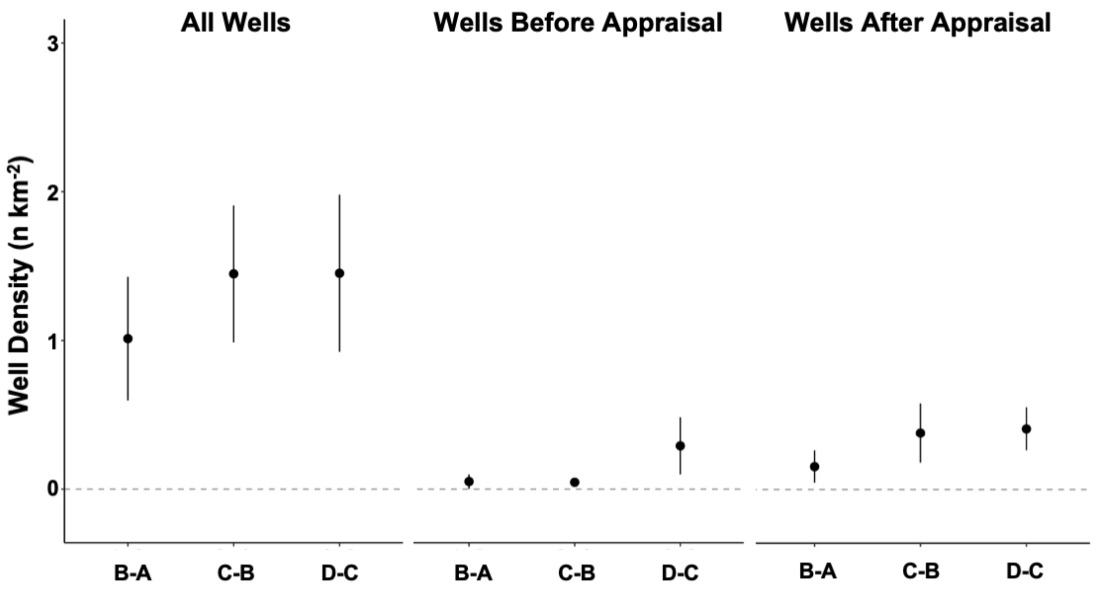

Figure S8. Point estimates and 95% confidence intervals for the difference in well density (number of wells per km^2) within 100 m of neighborhood boundaries, comparing neighborhoods with adjacent HOLC grades. These points represent the estimated increase in well count for neighborhoods with the relatively worse HOLC grade compared to propensity score-matched neighborhoods with the relatively better grade. We conducted separate analyses for: (a) all wells, including those without production dates; (b) wells drilled or operated before HOLC appraisal occurred in each city; and (c) wells drilled or operated after HOLC appraisal occurred. These results are similar to those reported in Figure 4, except for with well density rather than well count as the exposure metric.
